# Integrative Analyses of Uterine Transcriptome and MicroRNAome Reveal Compromised LIF-STAT3 Signaling and Progesterone Response in the Endometrium of Patients with Recurrent/Repeated Implantation Failure (RIF)

**DOI:** 10.1371/journal.pone.0157696

**Published:** 2016-06-15

**Authors:** Youngsok Choi, Hye-Ryun Kim, Eun Jin Lim, Miseon Park, Jung Ah Yoon, Yeon Sun Kim, Eun-Kyung Kim, Ji-Eun Shin, Ji Hyang Kim, Hwang Kwon, Haengseok Song, Dong-Hee Choi

**Affiliations:** 1 Department of Biomedical Science, CHA University, Seongnam, Korea; 2 Fertility Center of CHA Gangnam Medical Center, CHA University, Seoul, Korea; 3 Fertility Center of CHA Bundang Medical Center, CHA University, Seongnam, Korea; Michigan State University, UNITED STATES

## Abstract

Intimate two-way interactions between the implantation-competent blastocyst and receptive uterus are prerequisite for successful embryo implantation. In humans, recurrent/repeated implantation failure (RIF) may occur due to altered uterine receptivity with aberrant gene expression in the endometrium as well as genetic defects in embryos. Several studies have been performed to understand dynamic changes of uterine transcriptome during menstrual cycles in humans. However, uterine transcriptome of the patients with RIF has not been clearly investigated yet. Here we show that several signaling pathways as well as many genes and microRNAs are dysregulated in the endometrium of patients with RIF (RIFE). Whereas unsupervised hierarchical clustering showed that overall mRNA and microRNA profiles of RIFE were similar to those of endometria of healthy women, many genes were significantly dysregulated in RIFE (cut off at 1.5 fold change). The majority (~75%) of differentially expressed genes in RIFE including S100 calcium binding protein P (S100P), Chemokine (C-X-C motif) ligand 13 (CXCL13) and SIX homeobox 1 (SIX1) were down-regulated, suggesting that reduced uterine expression of these genes is associated with RIF. Gene Set Enrichment analyses (GSEA) for mRNA microarrays revealed that various signaling pathways including Leukemia inhibitory factor (LIF) signaling and a P_4_ response were dysregulated in RIFE although expression levels of Estrogen receptor α (ERα) and Progesterone receptor (PR) were not significantly altered in RIFE. Furthermore, expression and phosphorylation of Signal transducer and activator of transcription 3 (STAT3) are reduced and a gene set associated with Janus kinase (JAK)-STAT signaling pathway is systemically down-regulated in these patients. Pairwise analyses of microRNA arrays with prediction of dysregulated microRNAs based on mRNA expression datasets demonstrated that 6 microRNAs are aberrantly regulated in RIFE. Collectively, we here suggest that dysregulation of several major signaling pathways and genes critical for uterine biology and embryo implantation may lead to uterine abnormalities in patients with RIF.

## Introduction

Despite significant improvements in assisted reproductive technology (ART), a substantial numbers of patients undergoing ART fail to achieve successful pregnancy even after repeated attempts [1]. Failure to achieve a pregnancy following 2~6 *In Vitro* Fertilization (IVF) cycles with high-grade embryos transferred to the endometrium was defined as recurrent/repeated implantation failure (RIF) [2–4]. RIF still remains a major challenge for both clinicians and researchers to improve pregnancy outcomes in ART. Recently, many reports have suggested that local ‘endometrial injury’, such as endometrial biopsy or curettage prior to ART may improve the chance of embryo implantation in the endometrium of patients who suffer from RIF [3–5]. A recent meta-analysis reinforced improvement of clinical outcomes after endometrial injury prior to ART in these patients [6], although it is still controversial. In addition, intrauterine administration of human chorionic gonadotropin-treated autologous peripheral blood mononuclear cells (PBMCs) improves ART outcomes of women with RIF [7]. However, how these approaches may improve uterine environments with appropriate uterine receptivity in patients with RIF and what signaling pathways and/or genes are dysregulated in these patients remain largely unknown.

The endometrium reconstructs itself in each menstrual cycle to provide a favorable environment for blastocyst implantation [8–10]. Numerous factors including cytokines, growth factors, chemokines and adhesion molecules and their receptors have been suggested to participate in this complex process [10, 11]. Certain aspects of embryo implantation are considered similar between mice and humans, and thus, data obtained from diverse gene-manipulated mouse models have been used to extrapolate functional roles of these factors in humans [12–14]. However, the animal models still do have limitations to understand actual events of embryo implantation in humans. Thus, endometrial changes with uterine receptivity in humans have been persistently investigated even with strict ethical limitations. Several microarray experiments have been performed to obtain large-scale expression profiles of mRNAs and/or microRNAs in human endometrium during menstrual cycles [8, 9, 15–18]. They showed that expression patterns of many genes are dynamically changed during menstrual cycles. However, the lists of differentially expressed genes (DEGs) hardly overlap among these studies, suggesting that physiological changes of endometrium are far more complex than we assume [19]. Especially, molecular changes in the endometrium of patients with RIF (RIFE) which lead to implantation failure are largely unknown. Here we show that several signaling pathways including Leukemia inhibitory factor (LIF)-Janus kinase (JAK)-Signal transducer and activator of transcription 3 (STAT3) pathway as well as many genes and microRNAs are dysregulated in RIFE.

## Materials and Methods

### Patients and endometrial sampling

This study was approved by the Institution Review Board at CHA Bundang Medical Center, CHA University, before sample collection (IRB No 2011-01-001) and all women signed an informed consent form before participating in the study. The control group (n = 7) consisted of volunteer women under the age of 40 years with regular menstrual cycle, who had at least one normal pregnancy and delivery. Women who had a past record of infertility, those currently on oral contraceptive therapy and those with intrauterine contraceptive devices were excluded. The RIF group consisted of patients who had undergone at least three IVF cycles with good quality embryos, but failed to conceive (n = 15). All participants were recruited from Fertility Center of CHA Bundang Medical Center, CHA University (Seongnam, Gyeonggi, Korea). Uterine cavity of control women and patients with RIF was examined by transvaginal sonography and their endometrial thickness was measured. Endometrial biopsies were collected using Pipelle de Cornier^®^ device (CCD Laboratories, Paris, France) on day 21 of the menstrual cycle (midluteal phase). Biopsied samples were immediately transferred to a research laboratory, and processed for snap frozen at −80°C for RNA extraction and/or to be embedded in paraffin for histological evaluation and immunohistochemistry.

### RNA extraction and reverse transcription

Total RNA was extracted from each specimen using Trizol Reagent (Invitrogen life technologies, San Diego, CA, USA) according to the manufacturer’s protocols. The purity and concentration of all RNA samples were examined by using a microspectrophotometer (ND-1000, NanoDrop Technologies, Roackland, DE, USA). Five and three endometrial total RNA samples were randomly selected from each group to be used for mRNA and microRNA array experiments, respectively. Two μg of each RNA sample was reverse transcribed using random hexamer primers (Promega Corporation, Madison, WI, USA) and M-MLV reverse transcriptase (Promega Corporation, Madison, WI, USA) in a final volume of 40 μl. Subsequently, cDNA samples were used as the template for PCR using the specific primers (S1 Table) as designed for realtime RT- PCR.

### Microarrays and data analyses with GSEA

Microarrays for mRNAs and microRNAs were initially performed with uterine total RNAs. Agilent Human genome 8 x 60 K arrays (Agilent Technologies, Santa Clara, CA, USA) and Affymetrix GeneChip^®^miRNA 3.0 arrays (Affymetrix, Santa Clara, CA, USA) were hybridized with appropriate cRNA probes at the core facility of GenoCheck (Ansan, Gyeonggi, Korea). The expression value and detection calls were computed from the raw data and Gene Set Enrichment Analyses (GSEA, version 3.7) was applied to interpret expression profiles from microarrays (Broad Institute, Cambridge, MA, USA). GSEA was originally developed to identify cohorts of genes whose functions are integrated into a certain biological process and/or signaling pathways [20]. Pathways were ranked according to the significance of enrichment, and the validation mode measure of significance was used to identify pathways of greatest enrichment.

### Quantitative realtime RT-PCR

Realtime RT-PCR was performed using the iCycler (Bio-Rad, Hercules, CA, USA). QuantiTect SYBR Green PCR reagents (Qiagen, Disseldorf, Germany) were used for amplification and results were evaluated with the iQ5^™^ Optical system software. The gene expression level was calculated using the relative quantification approach based on the ΔΔCt method and this value was then normalized to the relative amounts of an internal control, rPL19 cDNA. All PCRs were performed in duplicate.

### Immunohistochemistry

Immunohistochemistry for phosphorylated STAT3 (pSTAT3), estrogen receptor alpha (ERα) and progesterone receptor (PR) was performed on 5 μm sections of formalin-fixed, paraffin-embedded endometria as performed previously [21]. Paraffin sections were deparaffinized in xylene and hydrated in a series of graded ethanols. After a PBS rinse, the endogenous peroxidase activity was quenched on incubation for 10 min with 3% hydrogen peroxide. Sections were then incubated with blocking buffer (4% bovine serum albumin in PBS) containing 5% normal serum for 1 h at room temperature (RT), and incubated with primary antibody in the blocking buffer for 1 h at RT and overnight at 4°C. The primary antibodies were 1:400 anti-pSTAT3 (#MA5-11189, Thermo Fisher Scientific, Rockford, IL, USA), 1:50 ERα (#SC-542, Santa Cruz Biotechnology, Santa Cruz, CA, USA), and 1:200 PR (#PM-9102-S, Thermo Fisher Scientific, Rockford, IL, USA). After washing three times with PBS for 5 min, each section was incubated with 1:200 goat anti-rabbit IgG as the secondary antibody (Bio-Rad, Hercules, CA, USA) in the blocking buffer for 1 h at RT. Hematoxylin was used for nuclear counter-staining of the sections [22, 23]. Assessment of staining intensity and distribution of pSTAT3, ERα and PR was made using a modified semiquantitative analysis of HSCORE scoring system as described elsewhere [22, 23]. In all cases, 1000 cells/sample were evaluated by three independent observers.

### Statistical analysis

All experiments were repeated at least three times. Quantitative variables are given as means ± standard deviation. The data were analyzed for statistical significance with the Student's *t*-test. *p*-values <0.05 were considered statistically significant.

## Results

### Characteristic and hormonal profiles of patients with RIF

The mean age, body mass index, and basal serum levels of hormones were similar between control healthy women and patients with RIF (S2 Table).

### Overall mRNA expression profiles of RIFE are not distinctly different from those of healthy women

**Fig 1:**
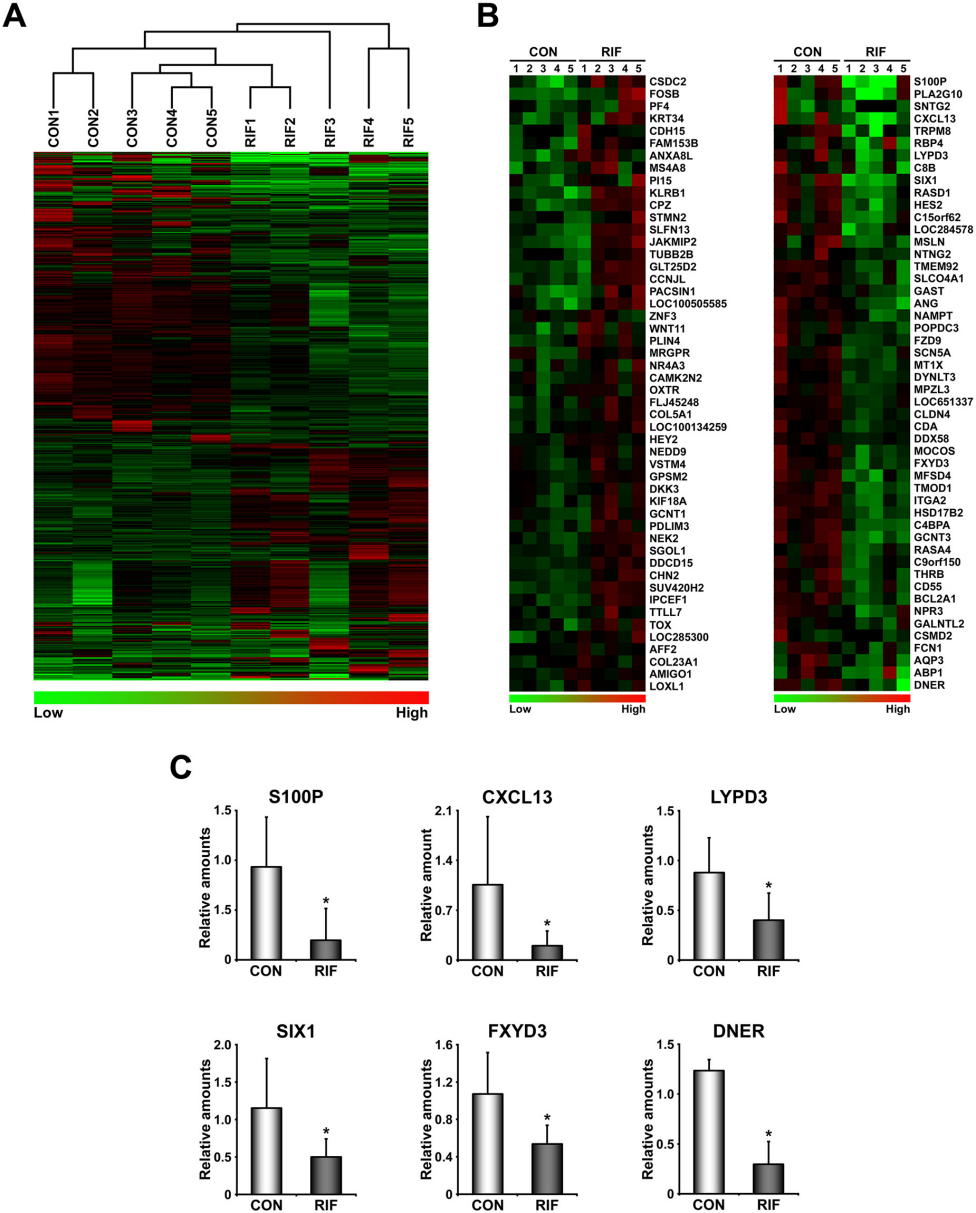
Global expression profiles of the endometrium of patients with RIF are not distinctly different from those of healthy fertile women. A) Unsupervised hierarchical clustering analysis for mRNA microarray data from endometria of healthy women and patients with RIF. B) Heatmaps for the 50 most increased and decreased genes in RIF. The color spectrum from green to red indicates low to high expression. C) Graphs of realtime RT-PCR results for genes whose expression is significantly reduced in RIF. CON and RIF represent endometrium of healthy fertile women and patients with RIF, respectively. *, *p*<0.05.

First we performed unsupervised hierarchical clustering for mRNA microarray data of endometria to determine whether overall endometrial transcriptome of patients with RIF is different from that of healthy women in midluteal phase. It shows that overall expression of RIFE is not distinctly different from that of healthy women (Fig 1A), suggesting that local signaling networks with genes critical for embryo implantation may be disturbed in RIFE. In fact, many genes are either up- or down-regulated (641 genes with 1.5 fold cut-off values) in RIFE (S3 Table). Of these, 164 genes (25%) were up-regulated and 477 genes (75%) were down-regulated, suggesting that DEGs in RIFE is mainly down-regulated. Fig 1B shows heatmaps of top 50 up- and down-regulated genes in RIFE. Realtime RT-PCR for S100 calcium binding protein P (S100P), Chemokine (C-X-C motif) ligand 13 (CXCL13), Ly6/PLAUR domain-containing protein 3 (LYPD3), SIX homeobox 1 (SIX1), FXYD domain containing ion transport regulator 3 (FXYD3) and Delta/Notch like EGF repeat containing (DNER) validated that these genes are significantly reduced in RIFE (Fig 1C).

### Identification of dysregulated signaling pathways in RIFE

**Fig 2:**
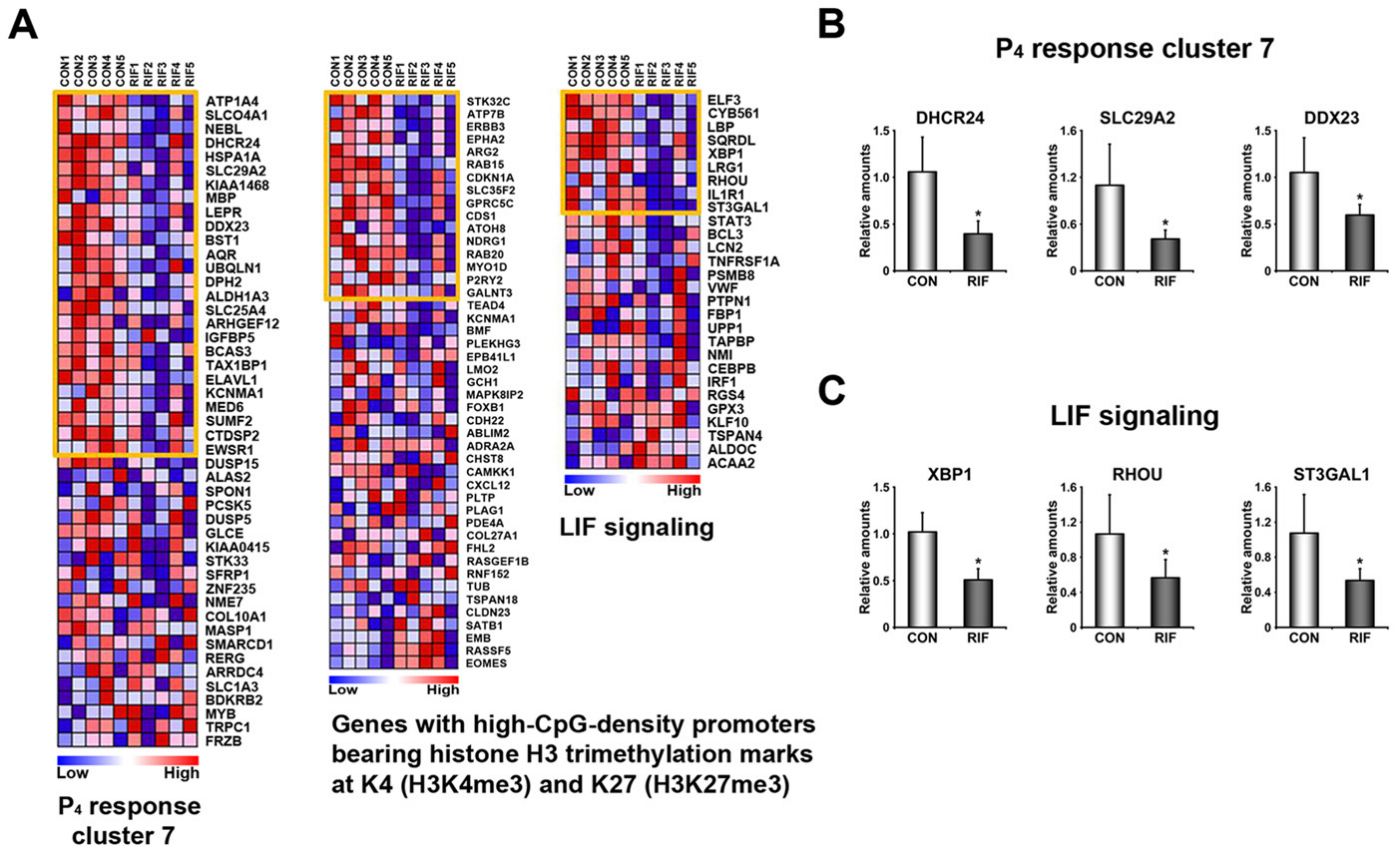
Dysregulated signaling pathways in the endometrium of patients with RIF. A) Heatmaps of representative gene sets that are aberrantly regulated in the endometrium of patients with RIF listed in Table 1. Genes within the orange boxes are leading characters for building enrichment scores in CON. The color spectrum from blue to red indicates low to high expression. B-C) Validation of systemic alteration of genes in these gene sets with realtime RT-PCR. CON and RIF represent endometrium of healthy fertile women and patients with RIF, respectively. *, *p*<0.05.

To gain insights into molecular causes leading to RIF in the endometrium, it is critical to identify significantly dysregulated signaling pathways and biological processes. GSEA, a supervised analysis program, was applied to provide insight into aberrantly regulated signaling pathways or biological processes in RIFE. The results suggested that various signaling pathways and biological processes are mainly reduced in RIFE (Tables 1 and 2). It is consistent with the result that 75% of DEGs are down-regulated in RIFE (S3 Table). Gene sets, such as epithelial_differentiation, metastasis and tumor_differentiated may be associated with poor differentiation of epithelial cells in RIFE. Furthermore, LIF signaling and a set of P_4_ response genes (Response_to_progesterone_cluster_7) are down-regulated in RIFE, suggesting that estrogen and progesterone actions may be impaired in these patients (Fig 2). Interestingly, a gene set for genes with high-CpG-density promoters bearing histone H3 trimethylation marks at K4 (H3K4me3) and K27 (H3K27me3) is dysregulated in the RIFE, suggesting that methylation profile may be also altered in these patients.

**Table 1:** The list of 22 down-regulated gene sets in the endometrium of patients with RIF.

Name	Size	Nominal P
OVARIAN_CANCER	51	0
BREAST_CANCER	44	0
HGF_SIGNALING	18	0
SILENCED_BY_TUMOR_MICROENVIONMENT	37	0
EPITHELIAL_DIFFERENTIATION	19	0
CEBPA_TARGETS	22	0
METASTASIS	87	0
OXIDATIVE_STRESS	17	0
TUMOR_DIFFERENTIATED	117	0
H3K4ME3_AND_HEK27ME3	26	0.011
ONCOGENIC_SIGNATURE	92	0
EGF_PERSISTENTLY	17	0.010
RESPONSE_TO_PROGESTERONE_CLUSTER_7	27	0.003
ENDOCRINE_THERAPY_RESISTANCE_3	227	0
IRF4_TARGETS_IN_MYELOMA	36	0
TARGETS_OF_SMAD2_OR_SMAD3	297	0
PALILLARY_THYROID_CARCINOMA	20	0.044
NRG1_SIGNALING	66	0.001
LIF_SIGNALING	18	0.011
RESPONSE_TO GONADOTROP	40	0.033
ADIPOGENESIS	28	0.049
RESPONSE_TOSALIRASIB	100	0.043

**Table 2:** The list of 3 up-regulated gene sets in the endometrium of patients with RIF.

Name	Size	Nominal P
ENDOMETRIUIM_CANCER	26	0.033
HYPOXIA	21	0.045
BREAST_CANCER_CLUSTER_1	16	0.010

### LIF-STAT3 signaling pathway is systemically dysregulated in RIFE

**Fig 3:**
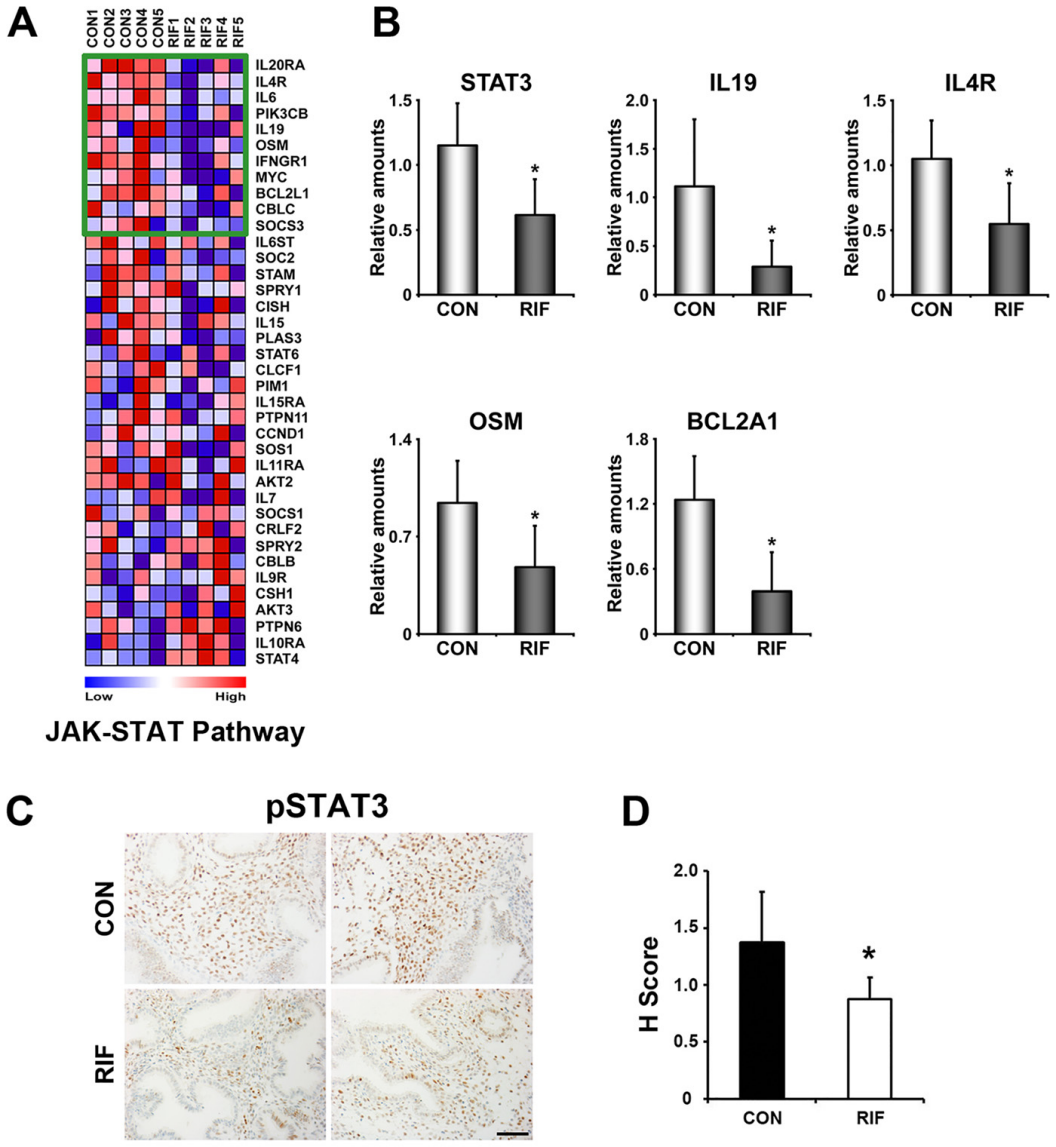
JAK-STAT3 signaling pathway is significantly reduced in the endometrium of patients with RIF. A) A heatmap of genes in the “JAK-STAT pathway” gene set. Genes within the green box are leading characters for building enrichment scores in CON. The color spectrum from blue to red indicates low to high expression. B) Realtime RT-PCR to confirm differential expression of genes within the green box of the gene set. C) Immunohistochemical analyses of phosphorylated signal transducer and activator of transcription 3 (pSTAT3) in RIF. Note that the number and intensity of STAT3 positive stromal cells were significantly reduced in RIF. D) HSCORE analysis for immunohistochemistry of pSTAT3 in endometrial stromal cells. CON and RIF represent endometrium of healthy fertile women and patients with RIF, respectively. *, *p*<0.05. Scale bar: 100 μm.

LIF is known as an essential factor of embryo implantation in mice and its expression in the human endometrium is also associated with uterine receptivity [24, 25]. Thus, dysregulation of LIF signaling in RIFE (Table 1) led us to examine whether STAT3, a major downstream effector of LIF signaling, is altered in these patients (Fig 3). Realtime RT-PCR results showed that STAT3 itself and a gene set associated with JAK-STAT signaling pathway including interleukin 19 (IL19), IL4 receptor (IL4R), Oncostatin M (OSM) and Bcl-2-related protein A1 (BCL2A1) are significantly reduced in RIFE (Fig 3A and 3B). Furthermore, immunohistochemistry of pSTAT3, an active form of STAT3, showed that STAT3 activity is notably lower in stromal cells of RIFE (Fig 3C). The HSCORE analysis for pSTAT3 reinforced a statistical significance in this comparison (Fig 3D).

**Fig 4:**
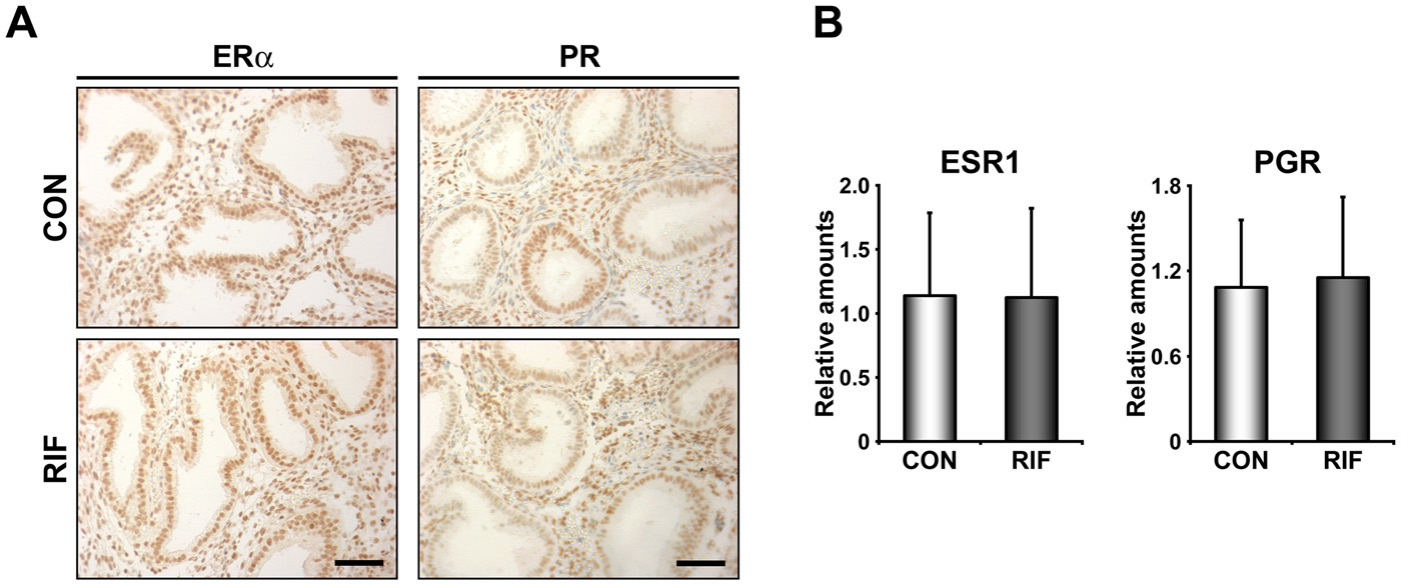
Expression levels of steroid hormone receptors are not altered in the endometrium of patients with RIF. A) Immunohistochemistry of estrogen receptor α (ERα) and progesterone receptor (PR) in the endometria of fertile control women (CON) and patients with RIF (RIF). B) The graphs of realtime RT-PCR results to compare relative expression levels of ERα (ESR1) and PR (PGR) between CON and RIF. Scale bar: 100 μm.

LIF is an established estrogen-responsive gene in the uterus [26, 27]. Since LIF signaling and a P_4_ response gene cluster are dysregulated in these patients (Fig 2), we examined whether ERα and PR are differentially regulated in RIFE. Realtime RT-PCR, immunohistochemistry for both ERα and PR, and HSCORE analyses for both receptors (Data not shown) collectively showed that there is no significant difference in their expression between healthy control endometrium and RIFE (Fig 4).

### Identification of dysregulated microRNAs in RIFE

**Fig 5:**
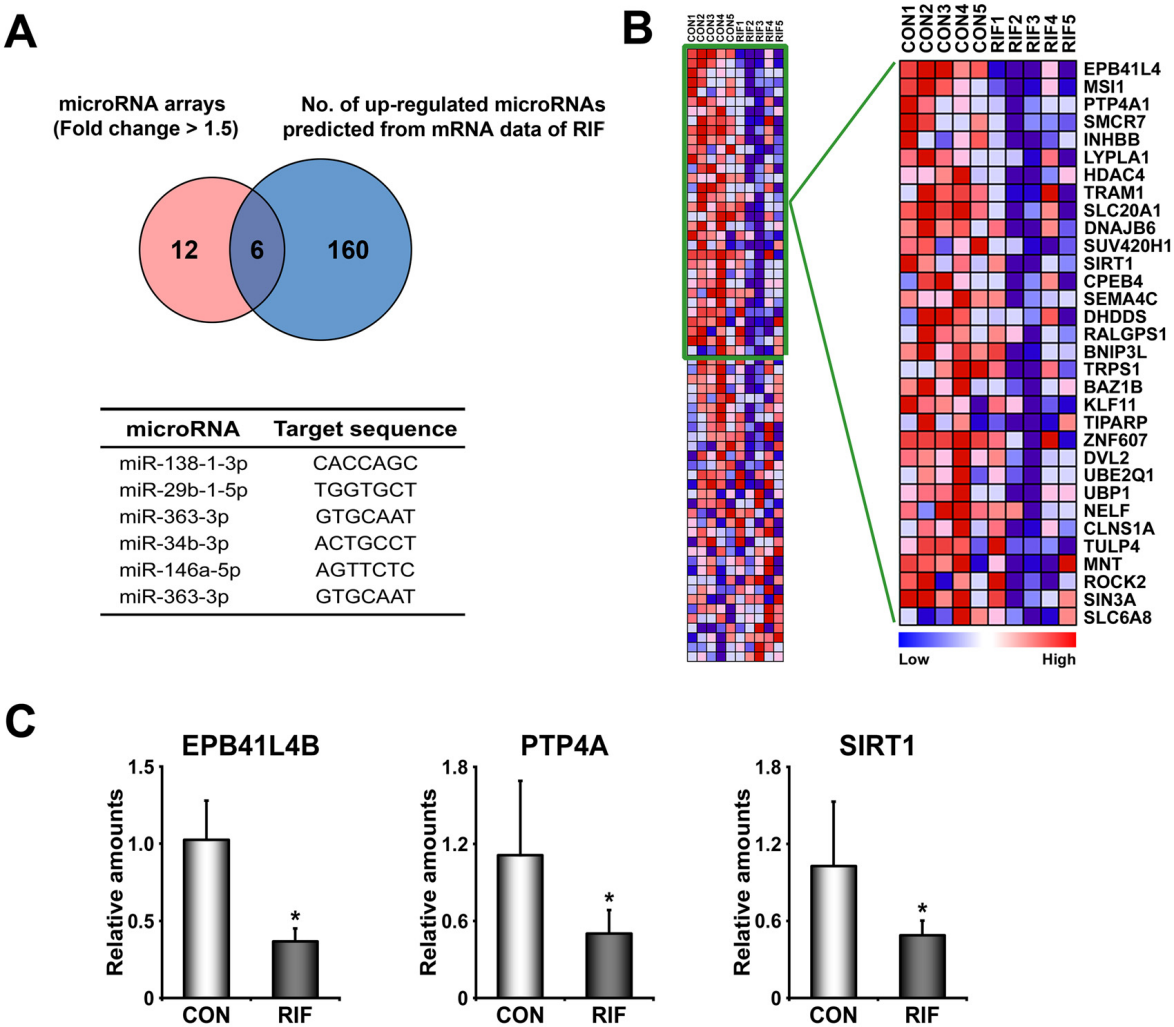
Dysregulated microRNAs in the endometrium of patients with RIF. A) A Venn diagram to show the number of up-regulated microRNAs collected from microRNA arrays and mRNA arrays in the endometrium of patients with RIF. MicroRNA arrays provided a list of 18 differentially expressed microRNAs, and GSEA with total expression profiles of mRNAs predicted 166 putative microRNAs. Six microRNAs (as listed in a table at the bottom) are overlapped between these two microRNA lists. B) A heatmap of putative target genes of miR-138-1-3p analyzed by GSEA. Genes within the green box are leading characters for building enrichment scores in CON. The color spectrum from blue to red indicates low to high expression. C) The results of realtime RT-PCR to validate down-regulation of putative target genes of miR-138-1-3p which is one of significantly up-regulated microRNAs in RIF. CON and RIF represent endometrium of healthy fertile women and patients with RIF, respectively. *, *p*<0.05.

In addition to dysregulated signaling pathways and genes, microRNA arrays provided a list of microRNAs whose expression is either increased or decreased in RIFE (S4 Table). GSEA analyses with mRNA expression data sets (transcriptome) predicted that 166 microRNAs could be aberrantly up-regulated in RIFE. Pairwise analyses for transcriptome and microRNAome for RIFE showed that 6 up-regulated microRNAs (miR-138-1-3p, miR-29b-1-5p, miR-363-3p, miR-34b-3p, miR-146a-5p, and miR-363-3p) are overlapped by these two analyses (Fig 5A). Among these microRNAs, we have validated putative target genes of miR-138-1-3p in RIFE. Collective down-regulation of putative target genes of miR-138-1-3p suggests that reduced expression of these genes in RIFE may be affected by overexpression of these microRNAs (Fig 5B and 5C).

## Discussion

Among the various potential causes of RIF, uterine factors (e.g., aberrant endometrial receptivity and immunological incompatibility) have become a recent focus of interest in ART [11, 28]. Endometrial receptivity could be modulated by a multitude of signaling molecules, including prostaglandins, growth factors, cytokines and chemokines [29, 30]. We showed that significantly down-regulated genes in RIFE including CXCL13 and S100P may be associated with poor embryo implantation in RIFE (Fig 1). It was suggested that CXCL13, a regulator of mucosal immunity, is abundantly secreted by human endometrial epithelium and specifically taken up by embryos with high implantation potential suggesting that it may be involved in embryo implantation [31]. S100P belongs to the EF-hand superfamily that mediates Ca^2+^-dependent signal transduction for cell growth, differentiation and metabolism. It is highly expressed during the implantation window in human endometrium [32, 33] and specifically induced in subluminal stromal cells surrounding the implanting blastocyst at the time of embryo implantation in mice [34]. Interestingly, S100P levels in natural cycle are higher than those in controlled ovarian hyperstimulation cycles [33], suggesting that it may be a marker for uterine receptivity in humans. The knockdown of S100A11, another S100 family member, in the uterus impaired embryo implantation in mice and it had adverse effects on the expression of factors related to endometrial receptivity and immune responses in human endometrial cells [35]. Furthermore, a recent proteomic analysis showed that S100A10 is up-regulated in midluteal phase and relatively down-regulated in the endometrium of infertile patients [36]. These reports collectively suggest that certain S100 family members may act as major players for establishing endometrial receptivity.

A variety of cytokines have been proposed to participate in sequential events of embryo implantation [37]. Especially, gene targeting experiments clearly demonstrated that IL6 family cytokines, such as LIF and IL11 are critical for these events [24, 38]. LIF deficient mice show complete implantation failure with no decidualization response [24, 26, 27]. Since then, the general significance of LIF signaling in embryo implantation has been reported in many species including humans. Uterine levels of LIF significantly increase around the period when the embryo initiates implantation in humans [25]. While LIF itself is not down-regulated in this study, STAT3, a major downstream factor to transduce signaling of LIF and other IL6 family in the endometrium, is down-regulated in RIFE (Fig 3). We further demonstrated that pSTAT3 is reduced in uterine stromal cells of these patients, suggesting that LIF-STAT3 signaling is aberrantly reduced in RIFE. In fact, it was reported that LIF is down-regulated in RIFE [39]. Significant reduction of LIF, LIF receptor (LIFR) and pSTAT3 was observed in endometrium of patients with dormant genital tuberculosis, suggesting a possibility that this infection could be one of reasons leading to RIF [40]. Furthermore, several studies showed that expression of LIF or its receptors, LIFR and Glycoprotein 130 (gp130), is reduced in the endometrium of patients with high risk of recurrent miscarriage and unexplained infertility [25, 41, 42]. Collectively, our results as well as previous studies strongly suggest that LIF-STAT3 signaling pathway is one of aberrantly regulated events which may cause RIF in these patients.

A previous study showed that ERα and a group of estrogen-dependent genes are systemically down-regulated in RIFE [43]. However, we did not find similar events with respect to expression levels and activities of ERα in RIFE (Fig 4). This discrepancy could be due to heterogeneous causes of RIF among patients. This assumption is also supported by the fact that DEGs in this study are quite different from those in the previous work [43]. Although expression levels and activity of ERα are not altered in RIFE, estrogen-dependent genes and signaling pathways could be locally impaired. In fact, we found that RASD1 and SIX1, significantly reduced genes in RIFE (Fig 1), are immediately induced by estrogen-ER pathway in mouse uterus (Kim et al., submitted) [44]. In addition, we suggest that P_4_ signaling is also locally impaired in RIFE while PR levels are not significantly altered in RIFE. Reduced expression of a set of genes whose expression is regulated by P_4_ (P_4_ response cluster 7) is consistent with the results of a previous study that P_4_ signaling is compromised in RIFE [19]. Impaired P_4_ signaling in RIFE is supported by the result that a set of genes associated with hedgehog signaling pathway, a well-known P_4_ downstream signaling, is dysregulated as well (S1 Fig). Considered that P_4_ is critical for the establishment and the maintenance of pregnancy by not only its endocrine but also immunological effects [45], RIF may be associated with immunologic imbalance caused by aberrant P_4_ signaling in RIFE. They collectively suggest that compromised P_4_ signaling is one of major causes for RIF since P_4_ supplementation could expand, to some extent, the length of uterine receptivity in mice [46] and improves clinical outcomes in humans [28].

Revel et al. suggested that a spectrum of microRNAs is either up- or down-regulated in RIFE [47]. While we also found that 6 microRNAs are differentially regulated in RIFE (Fig 5), none of differentially regulated microRNAs were overlapped with ones in the previous study. However, we found that biological pathways, such as JAK-STAT and Hedgehog signaling pathways, consisting of putative target genes of differentially expressed microRNAs in the previous study were also impaired in our patients with RIF (Fig 3 and S1 Fig). All these works strongly suggest that LIF-STAT3 and progesterone signaling pathways are aberrantly reduced in the endometrium of these patients. Pair-wise analyses for transcriptome and microRNAome with a large-scale number of endometrial samples of patients with RIF are required to further understand underlying molecular mechanisms by which uterine environments become impaired, leading to RIF. They may provide molecular evidence(s) for how injury-induced inflammation and/or administration of activated PBMCs improve uterine receptivity and the subsequent pregnancy outcome in patients with RIF [5, 48].

## Supporting Information

S1 Fig Hedgehog signaling pathway is down-regulated in the endometrium of patients with RIF. A) A heatmap of “Hedgehog signaling pathway” gene set. Genes within the orange box are leading characters for building enrichment scores in endometria of healthy women. The color spectrum from blue to red indicates low to high expression. B) Realtime RT-PCR results to validate differential expression of genes within the orange box of the gene set. CON and RIF represent endometrium of healthy fertile women and patients with RIF, respectively *, *p*<0.05.
(TIF)

S1 Table List of primers used in this study. (XLSX)

S2 Table Patients’ Characteristics (Clinical Data). (TIF)

S3 Table The list of up- and down-regulated mRNAs in the endometrium of patients with RIF. (XLSX)

S4 Table The list of up- and down-regulated microRNAs in the endometrium of patients with RIF. (TIF)
