# Supplementary figures and images for: Integrative Analyses of Uterine Transcriptome and MicroRNAome Reveal Compromised LIF-STAT3 Signaling and Progesterone Response in the Endometrium of Patients with Recurrent/Repeated Implantation Failure (RIF)
Source: PLoS One. 2016 Jun 15;11(6):e0157696. doi: 10.1371/journal.pone.0157696 (PMC4909214; doi:10.1371/journal.pone.0157696)

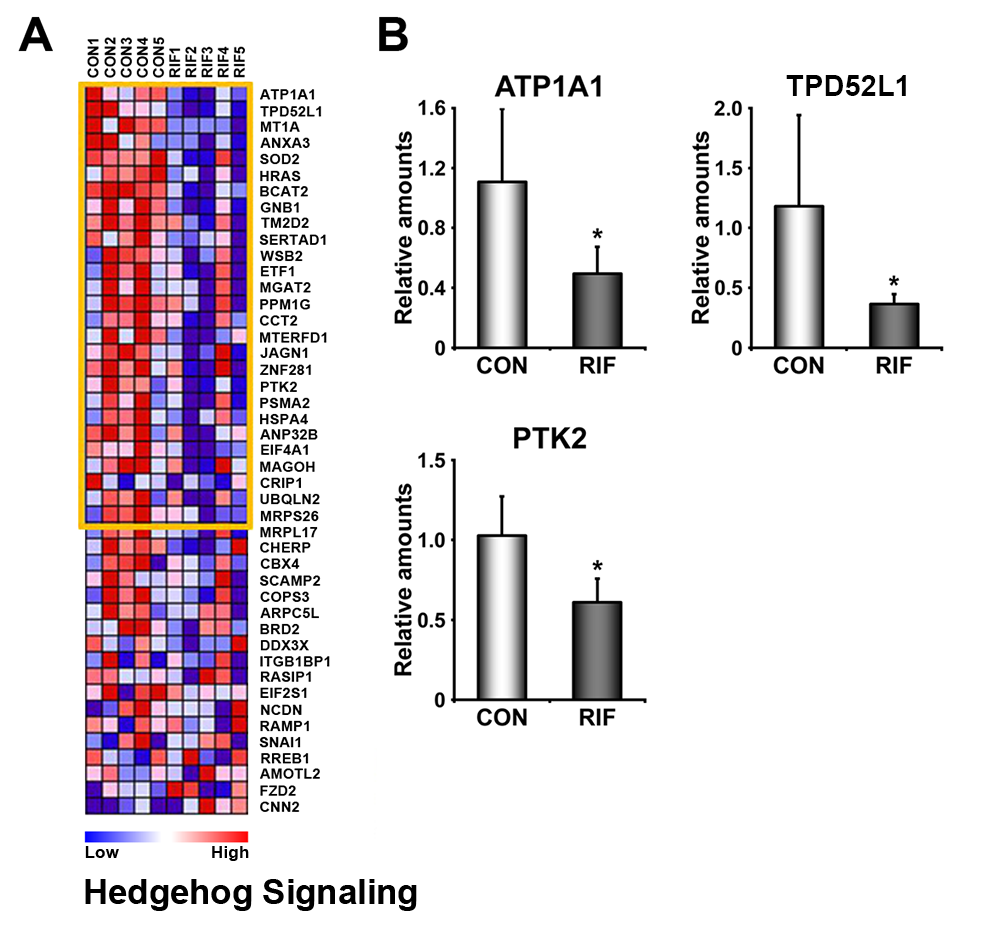

Supplement: S1 Fig — A) A heatmap of “Hedgehog signaling pathway” gene set. Genes within the orange box are leading characters for building enrichment scores in endometria of healthy women. The color spectrum from blue to red indicates low to high expression. B) Realtime RT-PCR results to validate differential expression of genes within the orange box of the gene set. CON and RIF represent endometrium of healthy fertile women and patients with RIF, respectively *, p<0.05. (TIF) [file pone.0157696.s001.tif]

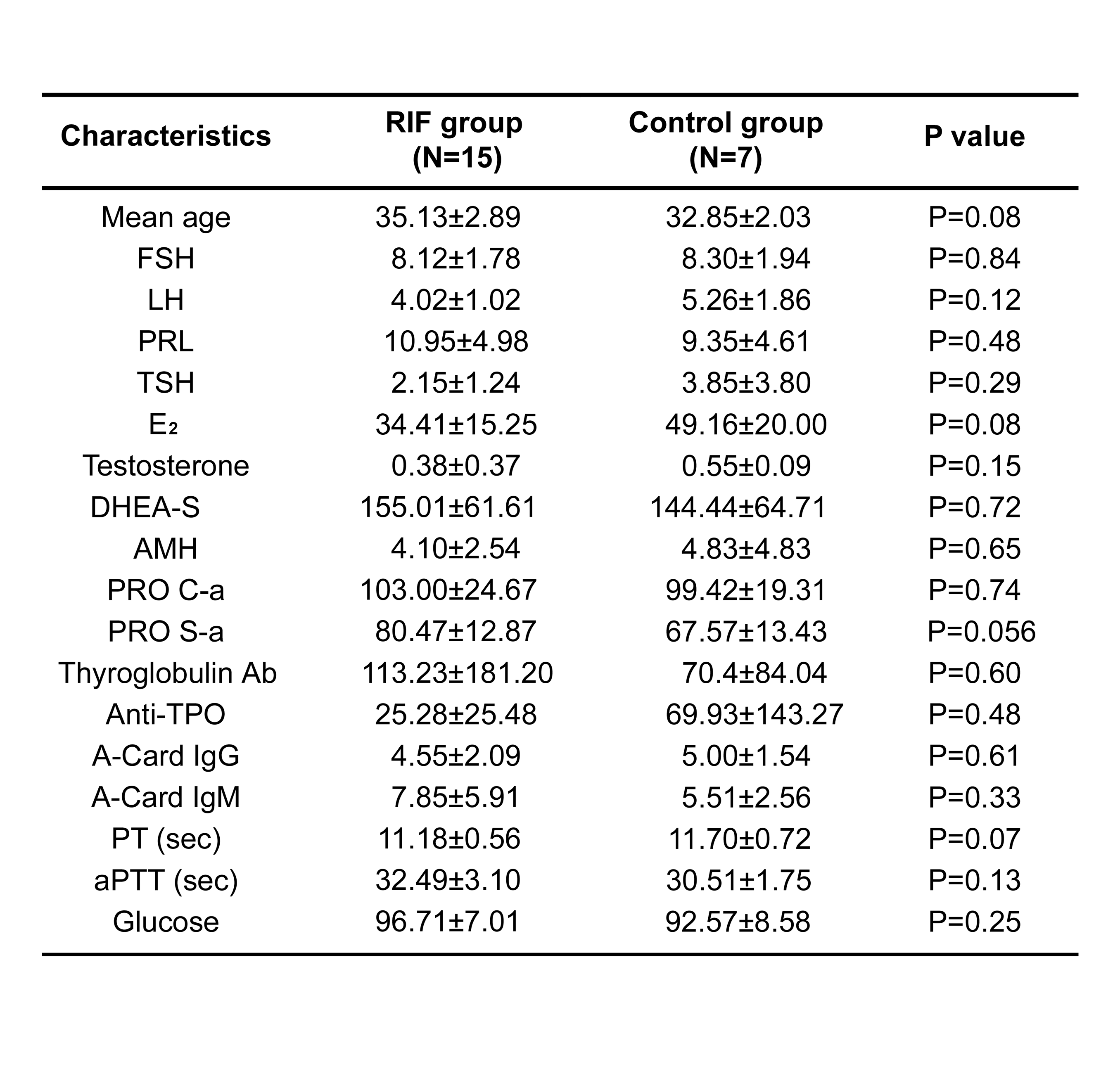

Supplement: S2 Table — (TIF) [file pone.0157696.s003.tif]

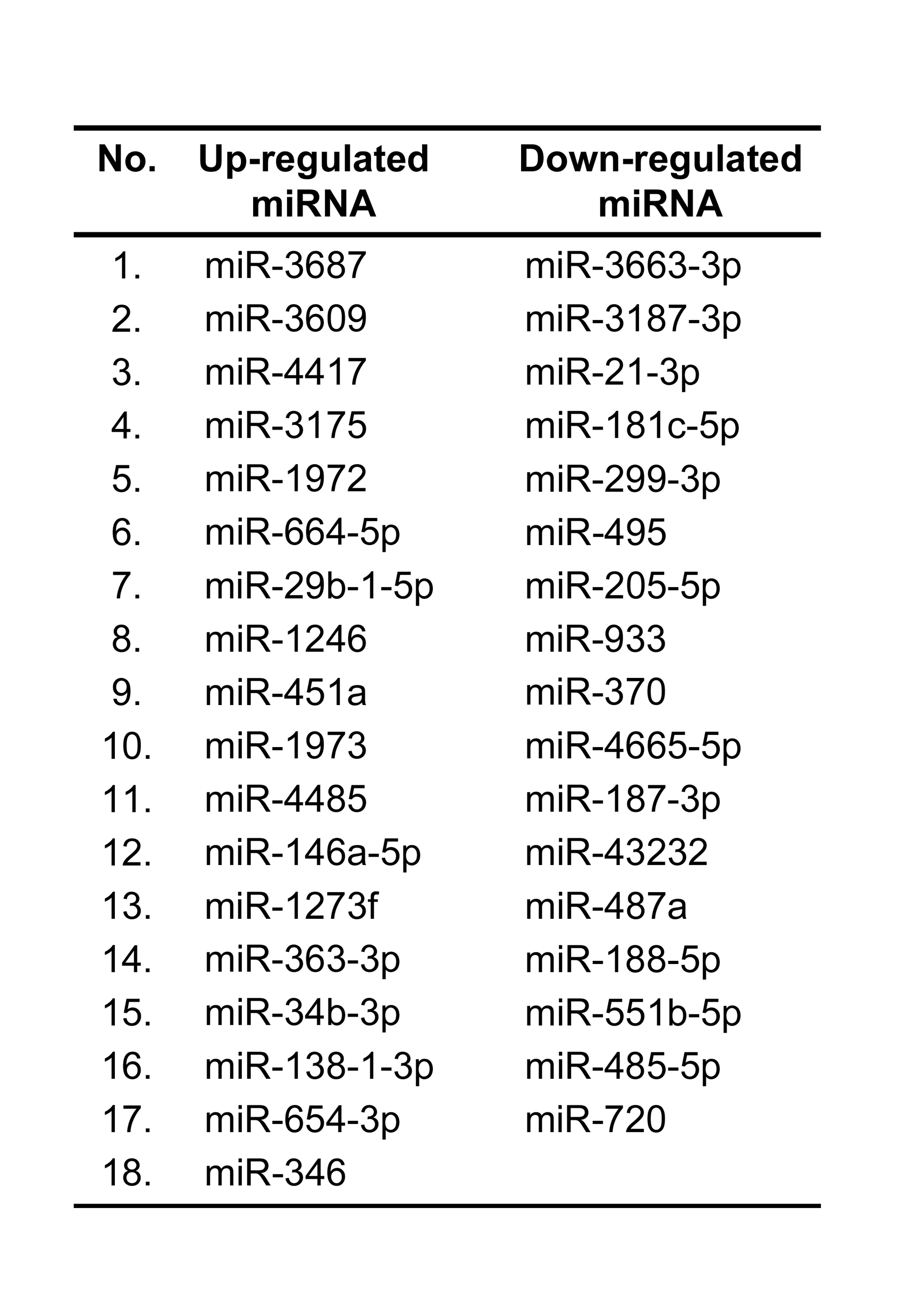

Supplement: S4 Table — (TIF) [file pone.0157696.s005.tif]
